# Combining Computational and Social Effort for Collaborative Problem Solving

**DOI:** 10.1371/journal.pone.0142524

**Published:** 2015-11-06

**Authors:** Mark D. Wagy, Josh C. Bongard

**Affiliations:** University of Vermont, Computer Science Department, Burlington, Vermont, United States of America; Defence Science & Technology Organisation, AUSTRALIA

## Abstract

Rather than replacing human labor, there is growing evidence that networked computers create opportunities for collaborations of people and algorithms to solve problems beyond either of them. In this study, we demonstrate the conditions under which such synergy can arise. We show that, for a design task, three elements are sufficient: humans apply intuitions to the problem, algorithms automatically determine and report back on the quality of designs, and humans observe and innovate on others’ designs to focus creative and computational effort on good designs. This study suggests how such collaborations should be composed for other domains, as well as how social and computational dynamics mutually influence one another during collaborative problem solving.

## Introduction

Machine intelligence is arguably out-competing humans in a growing number of domains: autonomous robots are taking over warehouse [1], construction [2], and agricultural [3] tasks; machine learning methods are becoming adept at finding patterns of interest in massive data sets [4] as well as heretofore unknown biological relationships [5] and even physical laws [6] in raw data; and new search methods are increasingly challenging professional players in complex games [7].

As this process spreads and accelerates, it remains to be seen what role humans will play in an increasingly automated society. We present evidence that one key role that humans may continue to play is one of cooperation with machine intelligence: complementing the speed of machines with the human capabilities of creativity, pattern recognition, and an ability to apply intuitions about the physical world to abstract problems.

So far it has been shown that casual users can help in scientific domains such as protein folding [8], galaxy classification [9] and brain analysis [10]. Additionally, human participants in crowdsourced experiments have contributed pattern recognition capabilities to algorithms for aiding algorithms in generating realistic images [11] and defining effective robot control schemes [12, 13]. However, the focus of these studies has been on the demonstration *that* it is possible to solve a complex problem by casual participants working collaboratively on the Web rather than understanding *why* such a group of volunteers is successful at these tasks.

Our hypothesis is that humans can effectively work as part of a hybrid human-algorithm team by contributing their experience as embodied and social organisms to their algorithmic counterpart. To test this hypothesis, we created treatments that combined human participants and search algorithms together in different ways. Each team was responsible for designing and programming autonomous robots such that the robots performed a desired task, which in this study was rapid locomotion.

We chose robotics as the domain in which to study human-computer collaboration as it has traditionally been viewed as an extremely challenging enterprise: only small academic or industrial teams composed of individuals with advanced degrees have so far produced capable robots. However, work on crowdsourcing robotics [12, 14, 15] has shown that it is possible for casual users to accelerate the programming of autonomous robots and provide them with some semantic understanding of the world [16]. These studies make clear that humans can play an important part in human-computer interaction and human-robot interaction, but an understanding of why and in what ways this collaboration can be successful is still under-explored.

Moreover, we here report the first investigation into whether casual users can design robots, not just help them learn: In some of the teams we created, participants were tasked with designing robots that their computer could then program to move rapidly. We hypothesized that people may be able to bring their intuitions about animal movement to bear on this problem: casual users may, consciously or otherwise, know what kinds of body plans facilitate (or obstruct) the discovery of fast forward locomotion [17–19].

## Methods

To understand how people and computers might work best together in this domain, we created three separate teams, each of which was tasked with designing and programming robots (note that we are here using the term “team” to distinguish between experimental treatments without necessarily referring to the standard social sciences definition of the term).

**Fig 1:**
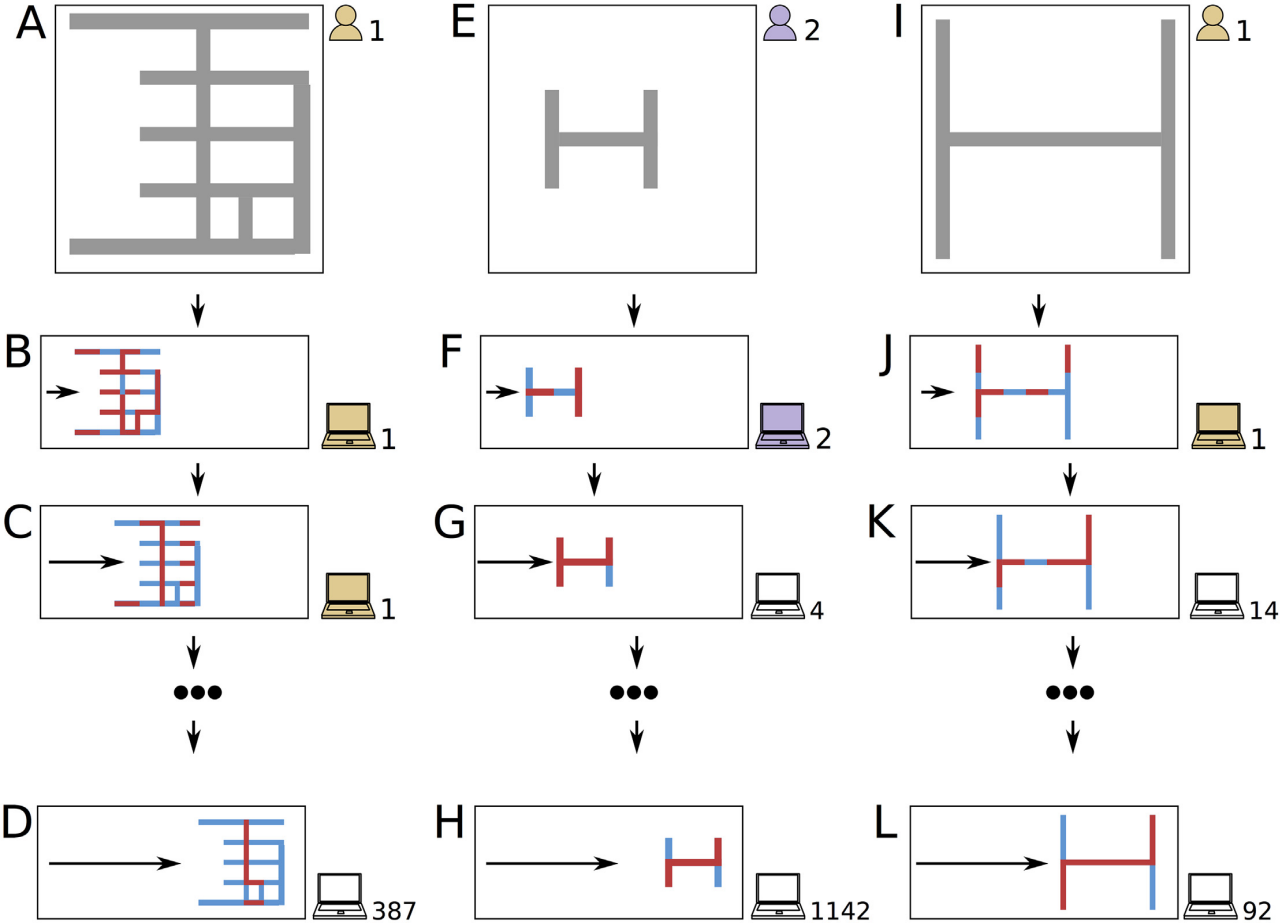
A hypothetical set of interactions in the crowd/machine team. Participant 1 designs a robot (a) and then allows an optimization method to program his robot once on his computer (b). (The red and blue body segments oscillate in anti-phase with each other, resulting in a small amount of forward travel indicated by the gray arrow.) Participant 1 allows the optimizer to re-program the robot (c), hoping its behavior will improve. Participant 387 sees and likes this design, so she allows the optimizer to try again on her computer (d). Meanwhile, participant 2 designs a different robot (e) and performs one round of optimization on it (f). Later, several more participants also contribute computational effort to this design (g,h). After observing the quality of participant 2’s design, participant 1 abandons his original design and attempts to improve on participant 2’s robot by creating a larger variant of it (i). After performing an initial round of optimization on it (j), several more participants confirm the quality of this design (k,l).

The first team was composed of human participants and algorithms: participants created, shared, and improved upon each other’s robot designs (Fig 1). [This work was exempted by the Committees on Human Subjects Serving the University of Vermont and Fletcher Allen Health Care, approval number 14–228.] Participants were recruited through the online bulletin board system, *Reddit*. We requested participation by querying users of several *subreddits*, pages devoted to subtopics of interest (such as artificial intelligence, robotics, programming and visualization). Therefore, participants were interested in technical subjects, but likely came from from varying backgrounds. However, users were anonymous so we cannot confirm the experience level of participants in the study with respect to each of these subjects. Reddit demographics are predominantly white, suburban males aged 18 to 29 years old [20]. All participants were unpaid volunteers.

The robots were virtual and behaved within a 3D simulated environment. Participants designed and observed their robots in a web browser (Fig 1a and were free to spend as much time at the task as they liked. They could also return and continue at any time. Each participant could also execute a search algorithm on their computer, which gradually improved controllers for their current robot (Fig 1b and 1c). The participant was free to dedicate as much or as little computational effort to a given robot as they liked, and could design as many robots as they liked. If the participant copied another participant’s robot design, the new participant’s computer continued searching from where the originating participant’s computer left off (Fig 1d. We will refer to this team as the *crowd/machine team* (CMT).

The second treatment was the same as the first, with one exception: participants could not see designs created by other participants. We will refer to this team as the *individual/machine team* (IMT).

### Robot design by the crowd/machine and individual/machine teams

Participants who arrived at the experiment website were double-blindly placed at random into either the crowd/machine team (CMT) or individual/machine team (IMT) with equal probability. By comparing the performances of the CMT and IMT, we were able to address the question of whether participants spontaneously collaborate in this domain. Do they improve upon promising designs created by their peers, or do they become mired in group pathologies such as groupthink [21, 22]? Although recent work has begun to quantify conditions under which teams work well [23, 24], social collaboration within human/machine teams requires further study. For both teams, robot designs were simulated using a web-embedded physics simulation engine (github.com/kripken/ammo.js/). The physics engine is a Javascript-based open source version of the popular C++ physics simulation engine, Bullet (http://bulletphysics.org/). The simulation was rendered using the WebGL graphics library (www.khronos.org/webgl/), a scene-based rendering library (http://scenejs.org/), and additional infrastructure code available online (schteppe.github.io/ammo.js-demos/).

**Fig 2:**
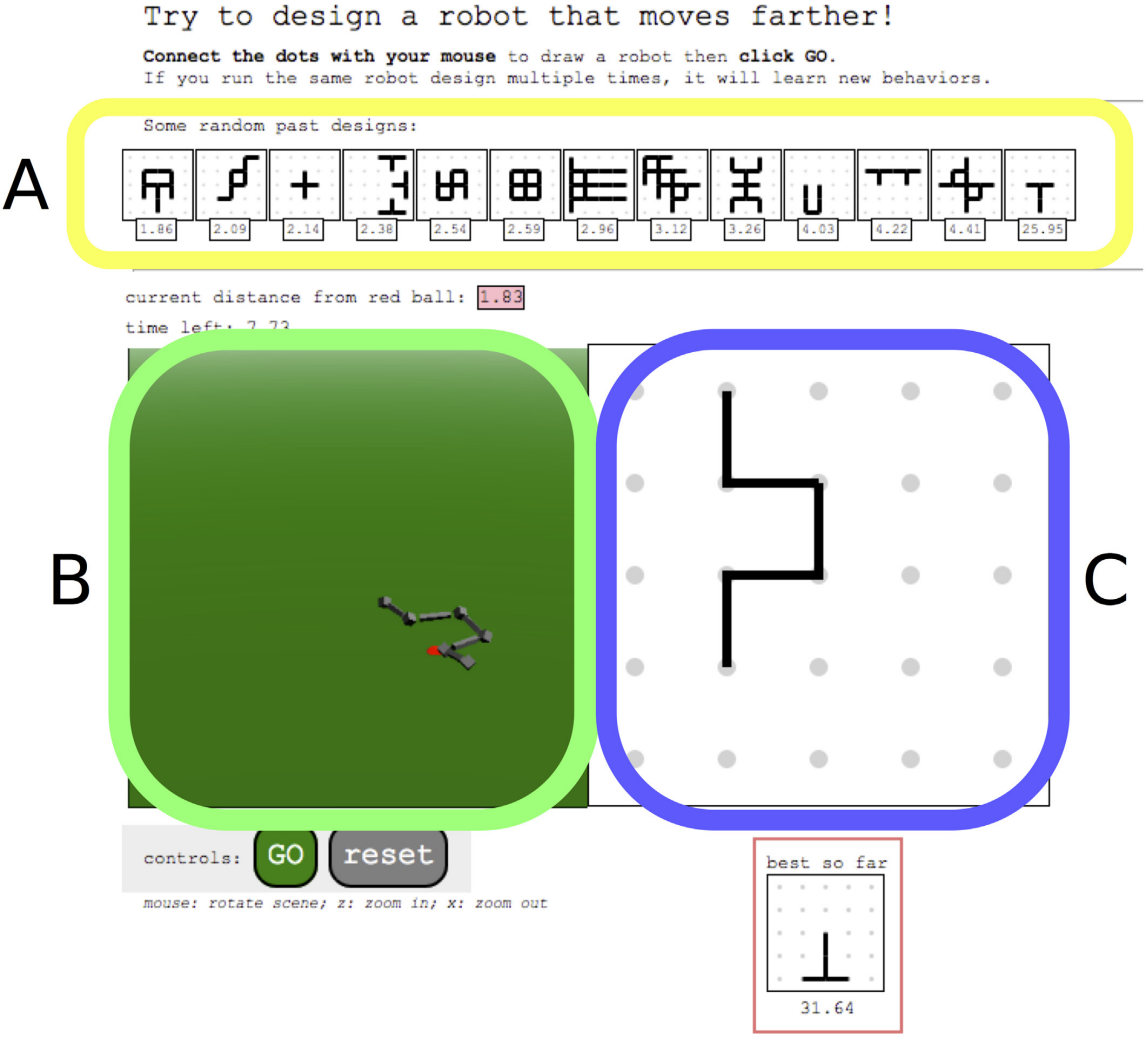
Screen-shot of the user interface with components. Enclosed in the yellow box is the history panel (a); in green, the simulation panel (b); and in blue, the design panel (c).

Each participant was instructed to design a robot that could move as far as possible in the simulation. Participants accomplished this by designing a robot in the design panel (Fig 2c), which was initially blank. They could then command a search algorithm to find good controllers for that robot. The quality of a controller is defined by how far it enables the robot to move from its starting position in fifteen seconds of simulation time. They could watch the progress of this optimization process in the simulation panel (Fig 2b). Members of the IMT could see their own past designs in the history panel (Fig 2a), while members of the CMT could see designs produced by themselves and other participants in the same panel. It was through this history panel that users ‘communicated’ designs to other participants.

Participants in either team were free to design as many or as few robots as they wished. They were also free to copy the designs produced by others (if they belonged to the CMT), create variants of other participants’ designs, or create completely new designs.

Participants could connect a 5-by-5 grid of points with lines. They could draw as many lines as the grid allowed. If the participant connected two points neighboring one another horizontally, or two points neighboring one another vertically, they were connected by a single line. If the participant attempted to connect two points that were not vertical or horizontal neighbors with positions (*x*_1_, *y*_1_) and (*x*_2_, *y*_2_), those points were connected with a series of horizontal or vertical lines connecting pairs of horizontally or vertically-neighboring points. These lines were chosen such that they approximate as closely as possible the straight line connecting (*x*_1_, *y*_1_) and (*x*_2_, *y*_2_). This made diagonal and/or overlapping lines impossible, simplifying the process of converting these points and lines into a robot that can be easily simulated in the rigid-body physics engine. They could click the ‘reset’ button at any time to re-start the design process.

Once the participant completed her design, the design would be rendered as a simulated robot using the physics engine. Each line of the design became a 1 × 0.1 × 0.1 rectangular solid, representing one part of the robot’s body. Each point adjacent to at least one line was instantiated as a 0.2 × 0.2 × 0.2 cube in the physics engine. The cube was attached to each neighboring body part by a one degree of freedom rotational joint. The axis of rotation for each joint was set perpendicular to the plane defined by a vertical line passing through the center of the cube and the horizontal line passing through the center of the cube and the center of the body part to which it attaches. This axis of rotation was chosen to allow the robot to move outside of the plane defined by the grid. Segments were able to push the robot away from the ground plane to achieve locomotion, thus making the morphology an important factor in its ability to move rather than relying only on the friction of a sweeping motion along the ground plane using each of its segments.

The interface was designed to be both easy to use and intuitive, as participants were not expected to have any prior experience designing robots or using the tool. The interface enabled participants to design robots simply by “connecting the dots”. Although this constrained design to a limited space of robots, the space was sufficiently large and sufficiently rich: there are 2^(5⋅4+5⋅4)^ = 2^40^ ≈ 1.1 × 10^12^ possible robots of differing sizes, symmetries, and biological realism, and which present varying levels of difficulty to the behavior optimization process. Each design produced by each participant of both teams was stored in a database on a central server.

### Behavior optimization for the crowd/machine team and individual/machine team

Once a participant completed a robot design, she could click the ‘go’ button, which would dedicate some of her own computer’s computational effort to finding a good controller for that design. When this button was pushed and the design did not yet exist in the central server’s database, a hill climbing search algorithm was assigned to that design.

The hill climber improved behavior for its assigned robot design as follows. The hill climber searched over the space of possible controllers for that design, attempting to find those that enabled the robot to move as far from its starting position as possible. Every time a participant pressed the ‘go’ button, one iteration of the hill climber would be performed.

The first time the participant pressed ‘go’ for a unique design she had created, the number of one-degree-of-freedom rotational joints in the robot’s design was counted. For a design with *j* such joints, a random binary string with *j* bits would be created. This string is assigned to the hill climber for this design. If a given design had received one or more iterations of search previously, then the current bit string associated with that design was copied, and each bit was flipped with 10% probability.

Then, the participant’s design was rendered as a simulated robot in the physics engine as described in the previous section. The robot was allowed to move in the simulator for 15 seconds and ∼22 cycles of motor oscillations. At each time step, each of the *j* motors associated with each joint is controlled with position control. The desired position sent to each motor is determined by a sinusoidal signal. This signal has a frequency of 1.5Hz and oscillates within [−45^*o*^,+45^*o*^]. All of the joints that have a zero associated with them in the current bit string oscillate in phase with one another, but are offset by a phase of *π* radians from those joints that have a one associated with them. The hill climber could in this way tune which joints move in phase or in antiphase with one another.

The participant could observe the resulting movement of the robot in the simulation panel (Fig 2). If the participant clicked the ‘go’ button again, her computer would perform another iteration of the hill climber. The more times a participant clicked the ‘go’ button, the more search would be conducted for that robot design.

Each bit string for every design was stored on a centralized server. So, if a participant drew a robot body that had already been attempted by themselves or another member of her group, her computer would perform another iteration of search for that design, starting from the best controller found up to that point. This enabled members of the CMT to consciously collaborate on a common design. However, participants in both groups could also unknowingly contribute computational effort to an existing design if they were not aware that that design had already been created by another member of their group: only 13 historical designs were shown to a participant in the interactive robot design tool but many more designs were stored on the central server. If a user invoked a design that had already been drawn and run by another user in her own group (either the IMT or CMT), she would be continuing the hill climber for that design.

Two databases on the central server were established: one stored designs and controllers from the IMT, and the other stored designs and controllers from the CMT. This ensured that members of one team could not continue behavior optimization for robots designed by members of the other group.

### Communicating the results of design and optimization

The top of the interface housed the history panel, which displayed 13 pictorial and numerical summaries of past robot designs. Members of the IMT could only see their own past designs. Members of the CMT saw their own past designs, as well as those produced by other members of their team.

Each summary presented a top-down view of the robot’s morphology, and the distance that that robot had managed to travel using one of the controllers that had been supplied to it by its hill climber.

Every time that a participant clicked the ‘go’ or ‘reset’ button, or refreshed the page, the 13 designs were erased. Each of the 13 slots was refilled as follows. For members of the IMT, one of their past designs was chosen at random. For the members of the CMT, any past design produced by members of that team was chosen at random. Then, one of the *n* controllers generated for that design by its hill climber was chosen at random from the central database. The robot design was drawn in the empty slot, and the distance traveled by that robot when controlled by that controller was written to that slot. The 13 summaries were then arranged in order, from the designs with the least displacement on the left to the most displacement on the right. This process was instituted to provide the participant with a sense for which morphologies may be amenable to controller optimization, and which morphologies may present the hill climber with a more multimodal search space.

Whenever a participant simulated a robot, its current displacement was reported in the simulation panel. If its current displacement matched or exceeded the distance achieved by a robot in the history panel, that number in the history panel would change from white to pink. This ‘thermometer’ metaphor gave the participant a real-time indication of how well their current design was able to move relative to the random sampling of past designs being displayed. It was hoped that this implicit competition might further incentivize participants to design robots with good potential for behavior optimization.

In addition to the thirteen randomly-sampled designs shown at the top of the interface, members of the IMT were shown their own best design at the bottom right of the page. The best design is defined as the one that had been most displaced by its hill climber, regardless of how many iterations of search each design has accumulated. Members of the CMT were shown the best design discovered by that team as a whole so far.

### Robot design by the machine team

The third team we studied was composed only of computers: a state-of-the-art search algorithm [25] was employed to design robot body plans and to find good controllers for them. It has been demonstrated [26–30] that this algorithm outcompetes other algorithms at designing and programming robots. We will refer to this team as the *machine team* (MT).

We selected the Compositional Pattern Producing Networks—Neuroevolution of Augmenting Topologies (CPPN-NEAT) algorithm [31] for this, as it has been shown to be superior to other search algorithms for finding efficient gaits for robots [29] and has also been employed for optimizing both robot morphology and control [26–28, 30, 32]. Another reason we chose this algorithm is because it was originally designed to mimic biological development’s bias towards the production of regular patterns. This bias toward regularity has been implicated in CPPN-NEAT’s ability to outperform other algorithms in automatically designing high-performance complex artifacts [31].

The CPPN-NEAT algorithm extends a simple evolutionary simulation, which at any point in time manages a population of Compositional Pattern Producing Networks (CPPNs). Each CPPN is capable of generating a regularly-patterned design within a user-defined, finite *n*-dimensional space. The user must also supply an objective function that can be used to score the quality of the resulting design. When the algorithm runs, CPPNs that produce designs with low objective function scores are deleted from the population, while CPPNs that produce high-performing designs are copied, randomly modified, and placed into the recently-emptied slots in the population. The algorithm continues for a user-specified number of generations. Finally, there are a number of experimental parameters that must be set before the algorithm can be run. For the work reported here we employed the CPPN-NEAT implementation available at www.multineat.com. We employed the same parameter settings as those used in [29].

Each CPPN generates a robot design as follows. Each CPPN takes four real-valued inputs and produces two binary outputs. From the five by five grid of points in the experimental website, each pair of horizontally neighboring points is selected in turn. For each pair, the horizontal and vertical coordinates of that point pair are input to the CPPN. The first binary output value is then read out. If the value is zero, the next point pair is supplied to the CPPN’s inputs. If the value is one, a line is constructed between those two points, just like a human participant might draw a line connecting that point pair. After each pair of 20 horizontally-neighboring points is supplied to the CPPN, the positions of each pair of 20 vertically-neighboring points is supplied to it. This resulted in the creation of zero or more horizontal and vertical lines connecting some of these point pairs together.

This process enabled each CPPN to design one robot. It was possible that some CPPNs create ‘null’ robots comprised of zero lines. Such robots were automatically assigned an objective function value of zero.

### Controller generation by the machine team

The CPPN created a controller for its robot as follows. For each CPPN, each pair of horizontally-neighboring and vertically-neighboring points connected by a line is iterated over. If each of these point pairs have positions (*x*_*i*_, *y*_*i*_) and (*x*_*j*_, *y*_*j*_), the CPPN is supplied with the position where one of the two resulting one-degree of freedom joints would be (*x*_*i*_, *y*_*i*_) and the position of the midpoint of the line connecting the point pair $\left(\frac{x_{i}+x_{j}}{2},\frac{y_{i}+y_{j}}{2}\right)$. The bit at the CPPN’s second output is then read out: a value of zero indicated that that motor would rotate the body segment centered at $\left(\frac{x_{i}+x_{j}}{2},\frac{y_{i}+y_{j}}{2}\right)$ about the cube centered at (*x*_*i*_, *y*_*i*_) with a phase phase offset of zero radians, while a value of one indicated that that motor would rotate with a phase offset of *π* radians. For the same point pair, the CPPN’s inputs were now supplied with positions (*x*_*j*_, *y*_*j*_) and $\left(\frac{x_{i}+x_{j}}{2},\frac{y_{i}+y_{j}}{2}\right)$. The bit arriving at the CPPN’s second output now dictated the phase with which the motor at (*x*_*j*_, *y*_*j*_) would rotate the body segment at $\left(\frac{x_{i}+x_{j}}{2},\frac{y_{i}+y_{j}}{2}\right)$ relative to the cube at (*x*_*j*_, *y*_*j*_).

The processes described in this and the previous sections thus enable a CPPN to generate both the morphology and control of a single robot.

### Behavior optimization for the machine team

When the CPPN-NEAT algorithm is initiated, it generates a population of *P* randomly-generated CPPNs. Each CPPN in the population is evaluated in turn. The CPPN produces its robot, the robot is evaluated in the physics engine, and the robot’s resulting distance from its starting position is assigned as that CPPN’s objective function score. Higher-scoring CPPNs are copied, mutated, and replace lower-scoring CPPNs in the population as described in [31]. The new entrants in this next generation of CPPNs are evaluated, and this process continues for a set number of generations. The number of generations was chosen so as not to exceed the number of evaluations that was reached by the CMT.

Because different variants of the CPPN-NEAT algorithm were conducted with differing population sizes, the number of generations *G* for each variant was set to the floor value of the number of evaluations performed by the CMT as a whole (11998) divided by the population size: $\lfloor \frac{11998}{P}\rfloor =G$. We performed 100 independent replicates of this algorithm and measured the average displacement of the robots produced by the best CPPNs in each population, at each generation.

In order to ensure the robustness of our results, we investigated the performance of the CPPN-NEAT algorithm using five sets of experimental parameters as reported in Table 1.

**Table 1:** Machine team experimental settings investigated.

Case	Population (P)	Mutation Rate	Representation	Generations (G)	Replicates
A	100	0.05	4 CPPN inputs	119	100
B	100	0.05	2 CPPN inputs	119	100
C	100	0.1	4 CPPN inputs	119	100
D	200	0.05	4 CPPN inputs	59	100
E	50	0.05	4 CPPN inputs	239	100

**Fig 3:**
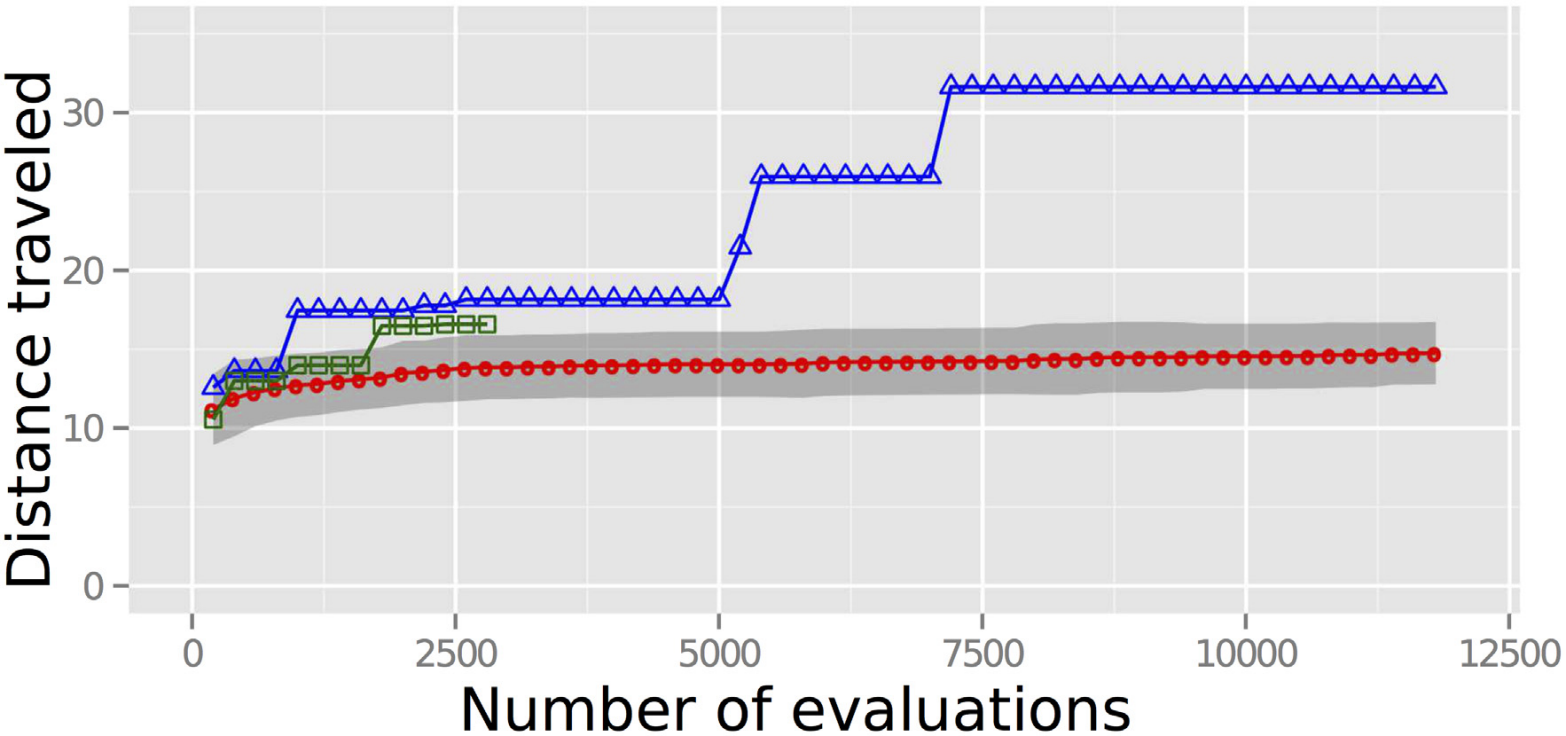
Comparing how the three treatments designed robots. The relative design abilities of the MT (red), IMT (green), and CMT (blue). The unit of distance *u* is set equal to the length of one robot body segment. The markers for the IMT and CMT report the furthest displacement achieved by any robot produced by that team up until that point. The markers for the MT report the mean displacement achieved by the 100 best robots, one drawn from each of the 100 independent trials of the search algorithm employed by this team. The gray band reports the 95% confidence interval for the MT.

Fig 3 reports the performance of the MT using the parameter settings in Case D. This set was reported because it achieved the highest mean performance of the five settings.

In addition to varying the experimental parameter settings for the CPPN-NEAT algorithm, we also investigated two different representations for the generation of robot morphology. We considered both a four-input representation described above as well as a two-input representation.

In the two-input representation, each CPPN has two instead of four inputs. To create a robot, the position of the midpoint between each of the 20 vertically-neighboring and 20 horizontally-neighboring grid points was input to the CPPN in turn. The output of the CPPN was interpreted in the same way as the four-input CPPN representation. The robot controller was created by inputting the coordinate of the midpoint between the center of the cube to which a given body segment was attached and the midpoint of that segment into the CPPN to determine whether the corresponding motor command had a phase offset of zero radians (an output of zero) or a phase offset of *π* radians (an output of one).

## Results

We found that robots designed by both human teams on average outperformed the designs created by the MT (Fig 3). Moreover, despite the fact that more computational effort was expended by the MT than by the CMT, no design generated by the MT was able to outperform the best design created by the CMT. Using the complementary cumulative probability distribution of MT performance at the final evaluation, we found that there was near zero probability that the MT would produce a better design than the CMT (probability less than 0.0001). The greatest distance achieved by a robot out of all runs and all treatments of the MT was 28.0 units as compared to the maximum distance of 31.7 achieved by the CMT.

Participation numbers for both the CMT and the IMT are summarized in Tables 2 and 3.

**Table 2:** Comparison of the aggregate behavior between two human/machine teams.

	**IMT**	**CMT**
Total evaluations	2839	2839
Total participants	367	384
Total designs	1246	1090
Average controllers per design	1.64 ± 2.17	1.67 ± 2.94
Designs per participant	4.56 ± 5.96	4.54 ± 4.99
Performance of the top 30 designs*	14.49 ± 2.79	16.48 ± 4.10
Perfectly symmetric designs (among the top 30 designs)	27	22

*Participants who collaborated produced superior designs compared to those who worked individually (Welch’s t-test; p = 0.036, DOF = 49.4, t = -2.16; random normally distributed independent samples)

**Table 3:** Comparison of the aggregate behavior between one of the human/machine teams and the machine team.

	**MT**	**CMT**
Total evaluations	11800	11800
Total participants	-	947
Total designs	1897 ± 118	2885
Average controllers per design	1.83 ± 0.08	2.40 ± 4.98
Designs per participant	-	5.63 ± 7.13
Performance of top designs	14.75 ± 1.01	31.64
Perfectly symmetric designs (among the top 30 designs)*	1	25

*The CMT produced significantly more perfectly symmetric designs than the MT.

**Fig 4:**
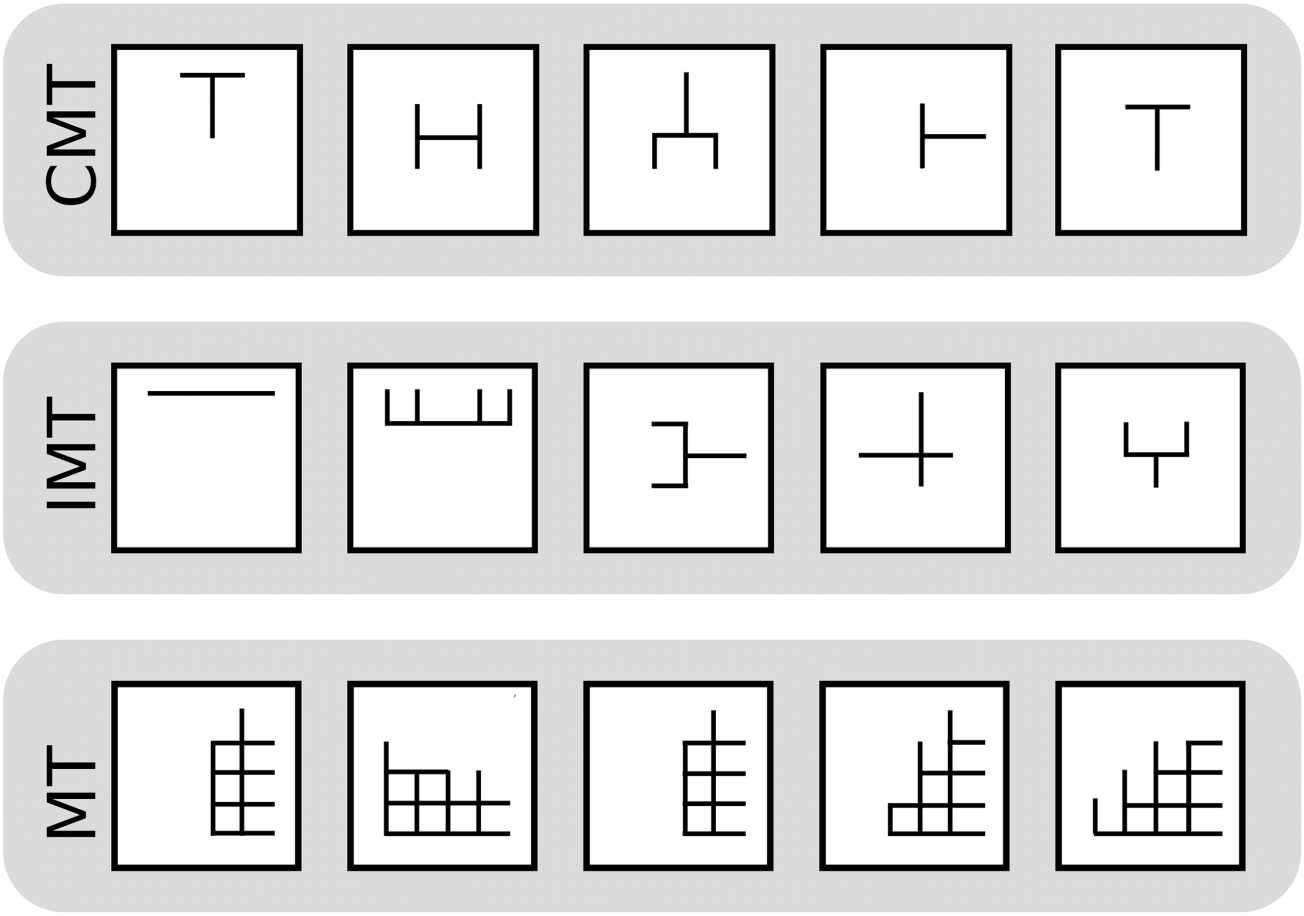
Top 5 robots in each of the treatments: the crowd/machine team (CMT), the individual/machine team (IMT) and the machine team (MT).

The top performing robots for each group are shown in Fig 4.

The individual/machine team portion of the experiment was stopped when we saw an appreciable decrease in participation (number of participants on day 1: 2805, day 2: 2108, day 3: 615, day 4: 164, day 5: 117, day 6: 3). The crowd/machine team was allowed to run longer for the sake of comparison to the machine team, giving the MT an opportunity to catch up to the CMT performance.

## Discussion

What caused the superior performance of the human/machine teams over the MT? The MT finds a robot that is able to achieve a distance of 28.0 units, which is near the best found by the CMT of 31.7 units, but this is the best result of 100 independent runs each of 5 different treatments explored with the MT: a much larger number of attempts than the CMT was allowed. Although the CMT evaluated more designs than the MT, we do not expect the superior performance to be due to these differences. This is because, in comparison, the IMT designed more robots than the CMT, yet performed worse than the CMT (Table 2). The MT did use CPPN-NEAT to define both the morphology as well as the control algorithm for the robots in contrast the the hillclimber that was used to define the control of the robots in the CMT and in the IMT. However, we hypothesize that using CPPN-NEAT for control versus defining the control scheme with a hill climber is in fact an advantage that the MT has over the human/machine teams based on past work [29].

In addition to creating what appear to be simpler designs (smaller number of segments and connections between them) as seen, for example, in Fig 4, a prominent feature of the designs created by the human/machine teams is the presence of symmetry (Table 3; see S1 Text for discussion of symmetry measure). Even though the search algorithm employed by the MT is known to be advantageous to other search methods of its type because it biases search towards more symmetric patterns [25], we observed a significantly higher number of designs that were perfectly symmetric among the top designs produced by the CMT compared to the top designs found by the MT. Moreover, human/machine teams focused on symmetric designs much more than would be expected, as there exist only two perfectly symmetric designs per million in the space of 1.1 × 10^12^ robot designs made possible through the user interface. Crowd participants focused on symmetric bodies at the outset of the experiment as well as the termination. This serves as evidence that the participants came to the experiment with a bias toward symmetric designs, even before being exposed to the performance of their own or others’ robot designs. The presence of perfectly symmetric designs created by participants was just as prevalent in the first 30 IMT designs as in the last IMT 30 designs: a significant number of these first designs were perfectly symmetric (23 of the 30, *p* = 0.0067; 1-sample proportions test, *χ*-squared = 7.50, df = 1; samples independently distributed); nearly the same number in the last 30 designs created by participants were perfectly symmetric (22 of the 30, *p* = 0.01762; 1-sample proportions test, *χ*-squared = 5.63, df = 1, samples independently distributed).

**Fig 5:**
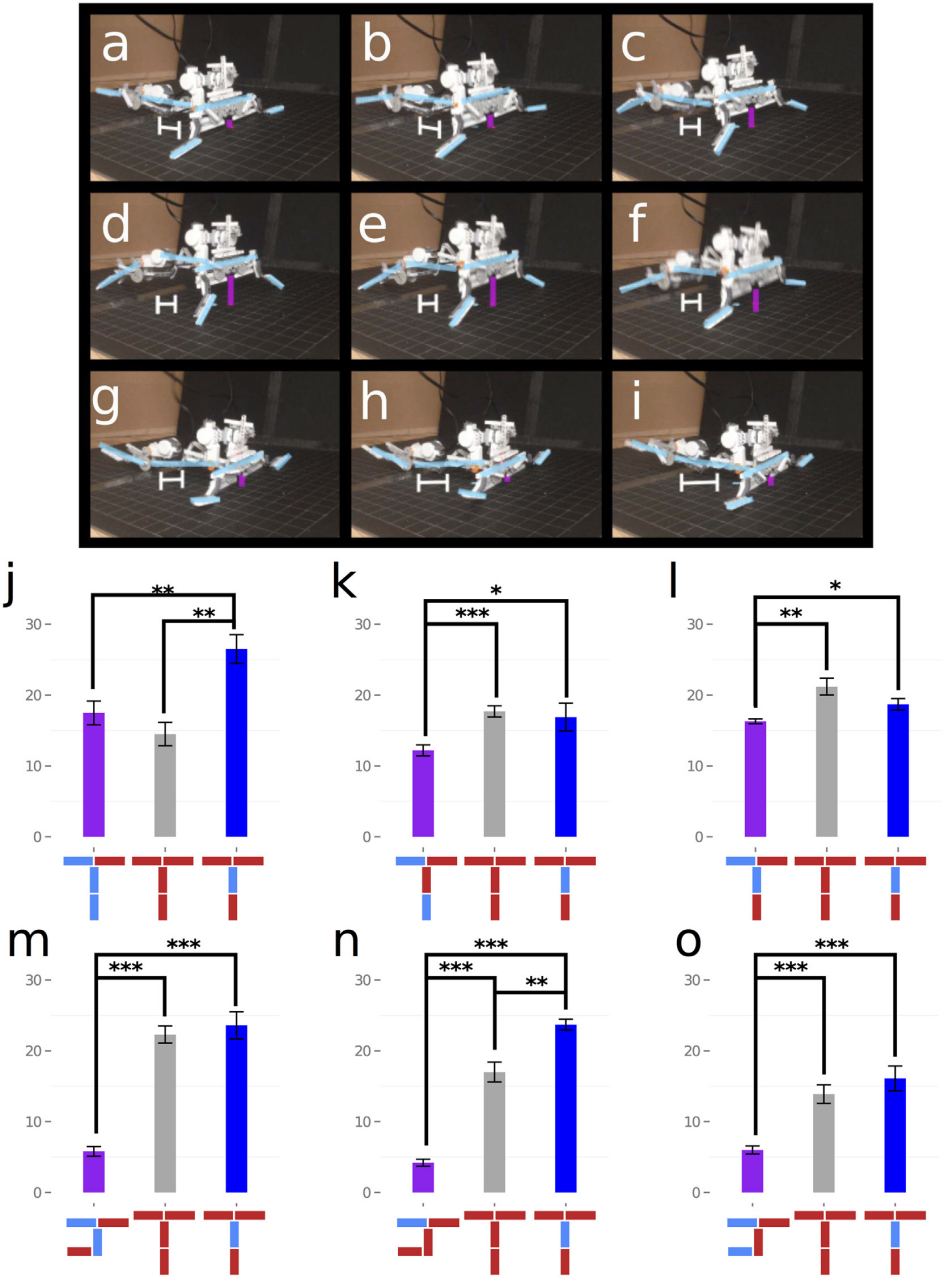
Performance of the physically constructed robot developed through the collaborative effort of 209 participants. **a–i**: gait of the physical realization of the best robot found by the CMT, and Panels **j–l** report the average distance travelled by the physical robot when equipped with the controller generated by the CMT (blue), three different randomly-generated asymmetric controllers (purple), and a randomly-generated symmetric controller (gray). Each bar in these panels reports average distance achieved over 10 independent trials. Panels **m–o** compare the average distance travelled by a randomly-generated, asymmetric, physical robot with the same topology as the symmetric robot (purple) to a randomly-generated symmetric controller on the symmetric robot body generated by the CMT (gray) and the symmetric controller discovered by the CMT (blue).

Bilaterally symmetric robot designs may be advantageous because, if coupled with a symmetric controller, they are better able to produce directed locomotion than bilaterally asymmetric robot body-plans, regardless of controller [33]. To test this we constructed a physical version of the best robot produced by the CMT, and ran it using the best controller found for that robot by the CMT, which also happened to be symmetric (Fig 5a–5i). We compared the performance of this physical robot against two variants: the same robot with randomly-generated asymmetric controllers (Fig 5j–4l), and an asymmetric robot composed of the same number of parts and controlled by randomly-generated asymmetric controllers (Fig 5m–5o).

We found that the symmetric controllers significantly outperformed the asymmetric controllers (Mann-Whitney U test with Bonferroni correction for multiple comparisons; [*p* = 0.0045, *p* = 0.0275, *p* = 0.0316], [*W* = 88, *W* = 79, *W* = 78.5], independent distributed samples) and, in all cases, the symmetric robot and controller outperformed the asymmetric robot with asymmetric controllers (Mann-Whitney U test with Bonferroni correction for multiple comparisons; [*p* = 0.0002, *p* = 0.0002, *p* = 0.0003], [*W* = 100, *W* = 100, *W* = 98.5], independent random samples). This suggests that symmetric robot body plans may be advantageous for this task, and that part of the explanation for why the CMT outperformed the MT is because its members were cognizant of this fact at some level of awareness, or became aware of it to some degree by viewing the quality of the designs produced by other members of their team.

In addition to a preference for symmetric designs, we investigated whether collaboration influenced the performance of the CMT. To do so, we compared the top designs produced by the CMT with those produced by the IMT.

This investigation was complicated however by the fact that varying numbers of robot body/controller combinations were evaluated by each of the CMT and IMT. In some cases, only a single controller was evaluated for a robot design, whereas other robot designs attracted many controller evaluations by members of a team. Thus, to better estimate the quality of robot designs produced by the IMT and CMT, we investigated in more depth how amenable to behavior optimization their best designs were. To do so, we first drew the top 50 ranking designs from both IMT and CMT according to the best controller found for those designs within their originating teams. We then performed 100 replicates of a genetic algorithm [34] against each design. Each genetic algorithm replicate was given a population size of 100 with a 10% mutation rate and 10% crossover rate. Genomes were bit-strings that indicated either a zero-phase (0) or *π*-phase (1) controller and the fitness objective was identical to that used in the web-based tool—a simulated robot was to move as far as possible within the allotted fifteen seconds of simulation time. The maximum displacement achieved by the design for each replicate was extracted and averaged over the 100 replicates. The resulting relative performance of the teams’ top *k* designs for various values of *k* is reported in Table 4.

**Table 4:** Distance moved by the top *k* robots after intensively optimizing their controllers. (Welch’s t-test; *p* < 0.05 for all values of *k*.)

**k**	**IMT distance moved**	**CMT distance moved**
20	14.4 ± 3.0	17.0 ± 4.1
30	14.5 ± 2.8	16.5 ± 4.1
40	14.7 ± 2.9	16.7 ± 4.1
50	14.6 ± 2.8	16.7 ± 4.1

Despite only slight differences between the aggregate behavior of the different teams (Table 2), the CMT’s best designs outperformed the IMT’s best designs (Welch’s t-test; *p* < 0.05 for all values of *k* [*k* = 20, *p* = 0.029, *DOF* = 33.3, *t* = −2.29;*k* = 30, *p* = 0.036, *DOF* = 49.4, *t* = −2.16;*k* = 40, *p* = 0.038, *DOF* = 51.3, *t* = −2.13;*k* = 50, *p* = 0.031, *DOF* = 50.8, *t* = −2.22], samples independent and normally distributed). The only difference between the two human/machine teams was that members of the CMT could see pictorial representations of their own and others’ designs, and the distances achieved so far by those robot designs, whereas members of the IMT could only see their own designs, and the distances those designs had so far achieved. This indicates that the superior performance of the social over the IMT must be due to collaborative innovation: members of the CMT avoided poor designs, dedicated more computation to promising designs, and/or created variants of good designs more than members of the IMT. In fact, the collaboration on designs can be seen in the average and maximum number of unique user contributions to designs in Table 5. Although groupthink and other group pathologies [35] may have been present in the CMT, their corrosive effects, if present, were outweighed by the positive feedback mechanisms of collaboration through non-verbal communication. This finding suggests that such collaboration may be harnessed in other design domains if appropriate pictorial representations, coupled with simple reports of the quality of each design, can be formulated.

**Table 5:** Unique user contributions to designs. The crowd/machine team resulted in more users contributing to individual designs than in the individual/machine team, suggesting that the crowd did contribute collectively to some designs.

	**CMT**	**IMT**
**Maximum Number of Users per Design**	210 *(out of* 919*)*	20 *(out of* 331*)*
**Maximum Proportion of Users per Design**	0.229	0.060
**Mean Number of Users per Design**	1.798	1.278

## Conclusion

Here we have shown that a team composed of human designers who can dedicate more or less computational effort to their own or other participants’ robot designs outperformed an machine team lacking human members as well as a treatment composed of human members who could not collaborate. Although we are not the first to demonstrate that human and algorithm teams can outcompete machine-only groups [8–10], we are the first to present reasons for why this occurs for design tasks. We showed that individuals bring pre-existing intuitions to bear on one part of the problem, while machines determine, through search, whether these intuitions are born out.

This intuition favored designs with symmetry, a morphological attribute that results in fast forward locomotion. That the crowd/machine team outperformed the individual/machine team demonstrates that social processes have a beneficial effect in this domain. While we cannot confirm which social processes resulted in the improved outcome, we hypothesize that this beneficial social collaboration derives from one or a combination of the following factors.

First, our interface allowed participants to view the work of others. We hypothesize that allowing participants to strengthen their pre-existing intuitions by viewing the work of others may have benefitted the crowd/machine team. Based on these growing intuitions, the participants could dedicate more or less computational effort to vet designs using search algorithms. The search algorithms in turn expose incorrect assumptions or validate intuitions. Second, human-produced designs and their machine-generated quality estimates were advertised to the group using a representation interpretable by casual users. If our interface did not represent the designs in this way, the improved performance of the crowd/machine team over the individual/machine team would have been possible only by chance, which we demonstrated to be unlikely (see Discussion section). Whether the participants benefitted from seeing the human-generated designs, the machine-generated quality estimates, or both is unknown, but at least one of the two must have been responsible for the superior performance of the crowd/machine team over the individual/machine team.

Thus, by capturing intuitions from casual users and exploiting synergistic social processes arising among them, we have shown that casual users may contribute useful creative work to a collaboration between humans and machines.

Given more time and computational resources, we would be interested in extending the analyses to account for other parameterizations of the evolutionary algorithms used in the study. Due to resource limits, we needed to define a somewhat arbitrary cutoff on the duration of the experiment and work with a fixed set of parameters in evolutionary algorithms. Had we investigated other parameters, we might have found results that were different from the findings of the present study. Additionally, the optimization method used may have converged upon a locally optimal result, thus missing the global optimum. It could be that the set of globally optimal robot designs are those that are not symmetric and the human participants exposed the algorithm to a locally, but not globally, optimal bias.

Future studies will focus on the social dynamics of the crowd interactions and the means by which human designers were influenced by both their interaction with other members of the crowd and their own experimentation with the design tool. In this study, we observed that it was beneficial for the human-algorithm variant to be exposed to other human designers. However, the modes that this social dynamic are beneficial may or may not be similar to the social dynamics present in other studies. Users may have, as the result of their own experimentation or by observing the design of other participants, discovered morphological variations that work well in tandem with a particular control structure; or they may have been able to improve substantially on a design by another participant that they might not have discovered on their own.

Additionally, vetting users according to their expertise would be an interesting addition the analyses. While we suspect that the majority of users in the present study were non-experts, the participants were anonymous and thus we cannot verify the extent to which the users were experts or non-experts.

Our finding suggests that increasingly large, diverse and complex collaborations that combine people and machines together in the right way (and further empowered by collaboration-enabling web tools, cost-effective additive manufacturing [36] and cultural trends such as the Maker movement [37]) may accelerate innovation in a wide range of fields. Finally, such work may help ensure that accelerating technological advancement develops into an empowering rather than a disenfranchising phenomenon.

## Supporting Information

S1 Video Demonstration and summary video. Video demonstrating user interaction and summarizing findings.
(MOV)

S1 Text Symmetry measure derivation. Derivation of measure used to report symmetry of robots in this article.
(PDF)
