# Supplementary material for: Combining Computational and Social Effort for Collaborative Problem Solving
Source: PLoS One. 2015 Nov 6;10(11):e0142524. doi: 10.1371/journal.pone.0142524 (PMC4636355; doi:10.1371/journal.pone.0142524)
Supplement: S1 Text — Derivation of measure used to report symmetry of robots in this article. (PDF) [file pone.0142524.s002.pdf]

Supporting Information, S1 Text: symmetry measure derivation

Completely symmetric designs in the space of robot morphologies possible with our characterization are few in number. The total number of robot designs possible on a 5-by-5 grid are $2^{(5\cdot 4+5\cdot 4)} = 2^{40} \approx 1.1 \times 10^{12}$. The number of bounding boxes (the smallest rectangular box that can enclose a given design) of d_1 grid units by d_2 grid units in a $D \times D$ grid is $(D - d_1 + 1)(D - d_2 + 1)$. Of each of these bounding boxes, we can have either vertical, horizontal or either of two diagonal lines of symmetry. The diagonal lines of symmetry were only considered if $d_1 = d_2$. The number of perfect horizontal symmetries are the number of designs that fit into one half of the bounding box that can be reflected about the vertical line of symmetry:

$$H_{sym} = \begin{cases} 2^{\lfloor \frac{d_1}{2} \rfloor (2d_2-1)} & : \ d_1 \text{ odd} \\ 2^{d_1(d_2-1)} & : \ d_1 \text{ even} \end{cases}$$

Similarly for vertical symmetry in the bounding box:

$$V_{sym} = \begin{cases} 2^{\lfloor \frac{d_2}{2} \rfloor (2d_1-1)} & : \ d_2 \text{ odd} \\ 2^{d_2(d_1-1)} & : \ d_2 \text{ even} \end{cases}$$

The number of symmetries for both diagonal axes when the bounding box is square is

$$D_{sym} = 2^{d(d-1)}, \text{ where } d = d_1 = d_2.$$

The total number of perfectly symmetric designs in a grid of size D are thus:

$$\text{Total \# symmetries} = \sum_{d_1=1}^{D} \sum_{d_2=1}^{D} (D - d_1 + 1)(D - d_2 + 1)(H_{sym} + V_{sym} + D_{sym})$$

For a 5-by-5 grid, the number of perfect symmetries (defined by reflections about an axis of symmetry in bounding boxes) is approximately 1.97 million. This amounts to ~ 1.82 perfectly symmetric designs per million designs in this space of all possible robot designs.
